# Supplementary material for: EzMechanism: an automated tool to propose catalytic mechanisms of enzyme reactions
Source: Nat Methods. 2023 Sep 21;20(10):1516–22. doi: 10.1038/s41592-023-02006-7 (PMC10555830; doi:10.1038/s41592-023-02006-7)
Supplement: Supplementary file 1 — Scoring formula, validation tables and EzMechanism documentation. [file 41592_2023_2006_MOESM1_ESM.pdf]

nature methods

Article https://doi.org/10.1038/s41592-023-02006-7

EzMechanism: an automated tool to propose catalytic mechanisms of enzyme reactions

In the format provided by the authors and unedited

Table of Contents

Formula used by the prioritisation algorithm..2

Validation Tables..2

Description of the Web User Interface - Input Page..6

Description of the Web User Interface – Output Page...12

References 15

Formula used by the prioritisation algorithm

The formula used to calculate the score of each step (graph edge) used by the prioritisation function has two parts, the first uses the largest bond length of all the new bonds created in that step (max_bond_length).

$$distance\_score = (\max(4, \quad max\_bond\_length) - 3)^2$$

With this formula, bonds up to a length of 4 Å yield the same score of 1. It is assumed that any distances under 4 Å are equivalent since the accuracy of the model, built from a PDB structure and with automatic positioning of the substrate, is lower than this. Furthermore, even if we assume the model is strictly accurate, it is possible for parts of the substrate and the side chain of residues to move closer together to react. Bonds larger than 4 Å are penalised according to a quadratic function, so that the penalisation quickly increases for larger distances.

The second part of the prioritisation is a simple halving of the score for steps whose reaction centres overlap with the reaction centres of the overall reaction. This is a heuristic measure to favour parts of the catalytic space that are moving the reaction forward. It is especially important when the substrate or cofactor is large and contains other reactive chemical groups that are not involved in the reaction such as the phosphate groups in NADPH.

$$score = \frac{distance\_score}{2}$$

When choosing the next configuration to match the rules, these scores are used in conjunction with Dijkstra's algorithm, which finds the configuration with the shortest path (using the scores of each edge as the distance) to the reactant or product configuration. In this way, configurations along paths that have lower scores are preferably selected. Since the final distance calculation is a sum over the scores of all reaction steps that lead to that configuration and there is a minimum distance (score) of 0.5, short paths are also favoured (all else being equal).

Validation Tables

Table extracted from: https://www.ebi.ac.uk/thornton-srv/m-csa/EzMechanism/

Table SI-I. Validation results for enzymes that already exist in the database

M-CSA ID	Prediction ID	PDB_ID	Results	All Rules Shortest Paths Length | n | Depth	Own Rules Shortest Paths Length | n | Depth	Other Rules Shortest Paths Length | n | Depth	Comments
2	1	1tem	See results	5 | 32 | 27 - 342	6 | 28 | 17 - 382	5 | 32 | 27 - 342	
7	1	4aj3	See results	4 | 10 | 11 - 73	4 | 6 | 11 - 73	0 | 0 | -	
8	1	3c2v	See results	7 | 312 | 600 - 615	7 | 4 | 600 - 615	0 | 0 | -	Tweaked Model PDB: to put N in the right place (substrate is symmetrical)
9	1	5t8s	See results	3 | 2 | 2	3 | 1 | 2	0 | 0 | -	Protonated His.
10	1	1mka	See results	4 | 1 | 21	4 | 1 | 21	6 | 4 | 26	protonated AspB
15	1	4h0d	See results	3 | 2 | 2	3 | 1 | 2	3 | 2 | 2	
16	1	4h0d	See results	4 | 2 | 5	4 | 1 | 5	9 | 10 | 194 - 335	deprotonated product

17	1	1a9t	See results	3 | 1 | 5	3 | 1 | 5	4 | 2 | 67	same molecule involved in two parts of the rule changed tautomerization state of products
21	1	1pj2	See results	4 | 8 | 11 - 344	5 | 2 | 24 - 26	4 | 8 | 11 - 344	
23	1	1kia	See results	2 | 1 | 1	2 | 1 | 1	2 | 1 | 1	tweak: changed protonation state of substrate
24	1	1nzy	See results	5 | 2 | 19	5 | 2 | 19	0 | 0 | -	Protonated Asp
26	1	4i9t	See results	3 | 20 | 2 - 145	3 | 4 | 2	3 | 20 | 2 - 145	tweak: protonated one of the histidines
28	1	1djy	See results	3 | 17 | 2 - 562	3 | 3 | 83 - 562	3 | 14 | 2	tweak: put proton in phosphate in products. R groups to C and fix isotope Model not the best, big distances
29	1	1djp	See results	4 | 4 | 57 - 138	5 | 2 | 127 - 138	5 | 529 | 126 - 625	tweak: deprotonated amine and protonated aminoacid to be consistent with M29 in products
31	1	1lce	See results	6 | 1 | 446	6 | 1 | 446	0 | 0 | -	Changed protonation states of Cys and Asp. works but very large distances throughout (water)
32	1	1fro	See results	5 | 4 | 28 - 30	5 | 2 | 28 - 30	5 | 4 | 28 - 30	
35	1	1phk	See results	3 | 2 | 2	3 | 2 | 2	3 | 2 | 2	protonate substrate in products
36	1	1qq6	See results	4 | 16 | 7 - 9	4 | 2 | 7 - 9	0 | 0 | -	protonated one of the waters in the products
38	1	4du6	See results	11 | 132 | 5127 - 7975	11 | 132 | 5127 - 7975	0 | 0 | -	10 steps protonated two his
40	1	2paa	See results	2 | 2 | 1	2 | 1 | 1	2 | 2 | 1	
43	1	2qfp	See results	3 | 2 | 2	3 | 1 | 2	4 | 107 | 13 - 930	his not protonated at the end, active site not recycled. phosphate with two protons
44	1	1ew8	See results	3 | 39 | 2	4 | 2 | 4	3 | 39 | 2	Step 4 is a regeneration step.
48	1	4rht	See results	3 | 3 | 2	3 | 3 | 2	0 | 0 | -	
50	1	3uwq	See results	4 | 2 | 7	4 | 2 | 7	0 | 0 | -	
51	1	1os1	See results	3 | 1 | 2	3 | 1 | 2	3 | 1 | 2	
52	1	3q94	See results	4 | 1 | 4	4 | 1 | 4	4 | 1 | 4	fixed position of the hydroxide in the products conformation of 3q94 not good, Glu too far away
53	1	2gq3	See results	4 | 4 | 14 - 377	4 | 4 | 14 - 377	0 | 0 | -	protonated products
54	1	3m7w	See results	9 | 424 | 374 - 8653	10 | 8 | 1261 - 6550	0 | 0 | -	
55	1	1gu1	See results	4 | 6 | 7	4 | 1 | 7	0 | 0 | -	Fixed protonation of Tyr and Glu
57	1	3a8o	See results	5 | 5 | 28 - 177	8 | 16 | 65 - 88	0 | 0 | -	
58	1	5o5k	See results	3 | 4 | 2	3 | 2 | 2	3 | 2 | 2	
60	1	1ne7	See results	9 | 18 | 4063	9 | 18 | 4063	0 | 0 | -	8 steps
61	1	1bjp	See results	3 | 1 | 2	3 | 1 | 2	0 | 0 | -	fixed 2d drawing of n-terminal proline
65	1	5k7x	See results	6 | 13 | 31 - 625	6 | 3 | 31 - 625	0 | 0 | -	protonated his and deprotonated substrate
67	1	1pjc	See results	5 | 2 | 313	5 | 1 | 313	6 | 3 | 160	changed protonation state substrate

69	1	2qia	See results	3 | 1 | 2	3 | 1 | 2	3 | 1 | 2	
73	1	1dcp	See results	5 | 2 | 21 - 48	5 | 2 | 21 - 48	0 | 0 | -	protonated two histidines
74	1	1dam	See results	5 | 2 | 41 - 137	5 | 2 | 41 - 137	0 | 0 | -	
75	1	12as	See results	4 | 5 | 11	4 | 4 | 11	4 | 1 | 11	protonated ammonia
77	1	4ubv	See results	5 | 2 | 33	5 | 2 | 33	0 | 0 | -	
78	1	3csc	See results	4 | 2 | 9 - 67	4 | 2 | 9 - 67	0 | 0 | -	protonated products
79	1	1jho	See results	2 | 1 | 1	2 | 1 | 1	0 | 0 | -	fix aromaticity of substrate and which N binds protonated glu in products
80	1	2x75	See results	4 | 8 | 41 - 222	4 | 1 | 41	0 | 0 | -	protonated one of the histidines
81	1	4csm	See results	2 | 1 | 1	2 | 1 | 1	2 | 1 | 1	
83	1	1l8s	See results	2 | 1 | 1	3 | 1 | 2	2 | 1 | 1	protonated products
84	1	1b66	See results	7 | 8 | 5634	8 | 4 | 3992 - 4218	0 | 0 | -	
85	1	1ik4	See results	5 | 6 | 113 - 493	5 | 2 | 113	7 | 6 | 91 - 98	
86	1	4bg4	See results	3 | 10 | 2	3 | 2 | 2	0 | 0 | -	deprotonated cysteine protonated product
87	1	5ol4	See results	5 | 24 | 13 - 282	5 | 8 | 13 - 282	0 | 0 | -	
90	1	5utu	See results	0 | 0 | -	0 | 0 | -	0 | 0 | -	protonated asp137 nad to nad+
91	1	3h5q	See results	5 | 5 | 4 - 21	5 | 1 | 4	7 | 26 | 110 - 914	
95	1	1fui	See results	4 | 23 | 4 - 381	5 | 2 | 92 - 184	4 | 21 | 4 - 23	
96	1	1chm	See results	5 | 1 | 103	5 | 1 | 103	0 | 0 | -	deprotonated product protonated second h2o
97	1	4eg2	See results	5 | 2 | 9	6 | 1 | 26	5 | 2 | 9	
98	1	5kob	See results	4 | 3 | 3 - 12	4 | 1 | 3	0 | 0 | -	changed protonation state of two products
100	1	5jry	See results	5 | 2 | 46	6 | 8 | 28 - 30	5 | 2 | 46	deprotonated cys protonated glu protonated water in product (extra proton)

Table SI-II. Validation results for enzymes not previously described in the database

PDB	Enzyme Name, references, and main result	Comments
7buy	SARS-CoV-2 Protease [1,2]<br><br>Mechanism found	Correct mechanism found, with several possibilities regarding exact protonation changes and concerted/non-concerted steps. Another set of mechanisms is suggested that use the side chain of the Ser in the substrate as the nucleophile. Information for these reaction steps come from an enzyme with different reaction (EC) and fold (CATH domain).<br><br>Another experiment where both products are charged also leads to a positive result but, this time, the suggested mechanisms are longer by one step.

1c0a	Aspartyl-tRNA Synthetase [3]<br><br>Mechanism found	EzMechanism can find the correct single reaction step. Both substrates (ATP and Aspartate) are modelled from the same PDB ligand, which is the product of the reaction. The rule is seen in 12 other enzymes annotated in M-CSA, including other aminoacid-tRNA ligases, but also other molecules that bind ATP, such as Ubiquitin, biotin, NAD, and luciferin. Although one would not need EzMechanism to find the mechanism of such simple reaction, the identification of enzymes that catalyse a similar step (with similar overall reaction and fold in some cases but different in others), might still be useful, and provide opportunities for further studies.
3jwe and 3hju	Monoglyceride Lipase[4]<br><br>½ Mechanisms found	EzMechanism can find the correct mechanism of monoglyceride lipase (R->1P->2df->2s->P). The nucleophilic attack of Ser onto esters is common in lipases. While monoglyceride lipase is not specifically annotated in M-CSA, other lipases with the same fold and similar reaction are (rule seen in 30 other catalytic steps).<br><br>For the same enzyme, using 3jwe, we tried to find the mechanism of the covalent inhibition of monoglyceride lipase by SAR629 (see reference). EzMechanism could not find any viable mechanistic path for this reaction, since there are no examples in the database of a hydroxymethyl group attacking a carbamide (urea) group. There is, however, a nucleophilic attack of hydroxide onto urea (in urease - m87) which, however, does not match this active site exactly. The development of more generic rules could help in this case.
5foe	Protein O-fucosyltransferase 2[5]<br><br>Mechanism found	EzMechanism can find the correct two step mechanism. This is another case where the mechanism is easy to infer from the reaction, but the information captured by EzMechanism is still useful. The rule associated with the main nucleophilic attack is seen in other transferases that have different structural folds (m339 – CATH:3.40.50.2000, m570 and m801 – CATH:3.90.550.10) and completely different substrates, where the hydroxyl group of Serine is "replaced" in these other substrates by the hydroxyl groups of sugar molecules and a 5-hydroxymethyl deoxycytidine group.
1lw4	L-Threonine Aldolase[6]<br><br>Mechanism not found	EzMechanism cannot find the correct mechanism. We suspect that this reaction is too different from what is annotated in the database, which contains many enzymes with PLP but that catalyse different types of reactions, and also contains many aldolases but using different active sites.
5xh3	PET Hydrolase[7,8]<br><br>Mechanism found but not for the exact substrate	EzMechanism cannot find any mechanism for PET hydrolase when a large part of the substrate is included in the calculation. When trying the same calculation with a trimmed version of the substrate (excluding the aromatic rings) EzMechanism is able to find the correct mechanism (R->1m->2bh->3m->P) while also suggesting many other possibilities using rules from several types of esterases. The negative result in the first case is caused by the fact that the rule codifying for the Ser nucleophilic attack does not match the aromatic bonds of the substrate. Since the problematic bonds and atoms are on the second shell of the rule, away from the reaction

		centres, this problem might also be overcome by making the rules more generic.

Description of the Web User Interface - Input Page

The following text and pictures are also available at https://www.ebi.ac.uk/thornton-srv/m-csa/EzMechanism/documentation

Introduction

EzMechanism is a web tool able to automatically generate possible catalytic mechanisms for a given enzyme active site and reaction. It is a knowledge-based approach, which works by searching the chemical reaction space available to the enzyme, using a set of catalytic rules inferred from the mechanisms annotated in the M-CSA.

1 - Access to EzMechanism

EzMechanism is only accessible to registered M-CSA users. To register please send an email to ribeiro@ebi.ac.uk with the desired username and an account will be created for you. Automatic registration will be added in the future.

Once registered the user can log in with the provided credentials by clicking on the "Log in" button in the main navigation bar. After login, users will be able to access EzMechanism by clicking on the EzMechanism button which will then be visible.

The main EzMechanism page has a table with all the searches previously created by the user (example in the figure below). For new users, this table will be empty.

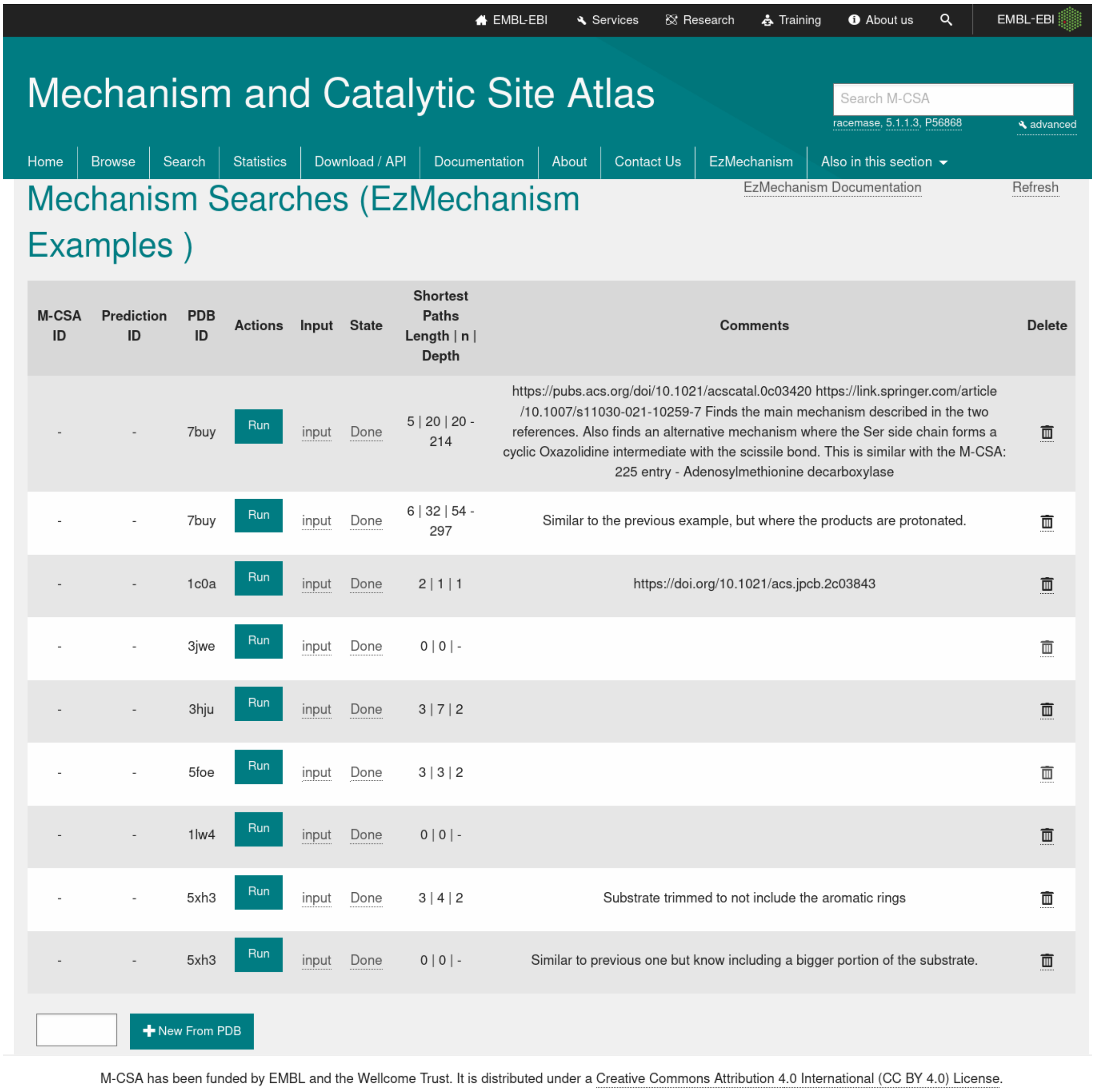

EMBL-EBI Services Research Training About us EMBL-EBI

Mechanism and Catalytic Site Atlas

Search M-CSA

racemase, 5.1.1.3, P56868 advanced

Home | Browse | Search | Statistics | Download / API | Documentation | About | Contact Us | EzMechanism | Also in this section

Mechanism Searches (EzMechanism Examples)

EzMechanism Documentation Refresh

M-CSA ID	Prediction ID	PDB ID	Actions	Input	State	Shortest Paths Length | n | Depth	Comments	Delete
-	-	7buy	Run	input	Done	5 | 20 | 20 - 214	https://pubs.acs.org/doi/10.1021/acscatal.0c03420 https://link.springer.com/article/10.1007/s11030-021-10259-7 Finds the main mechanism described in the two references. Also finds an alternative mechanism where the Ser side chain forms a cyclic Oxazolidine intermediate with the scissile bond. This is similar with the M-CSA: 225 entry - Adenosylmethionine decarboxylase	
-	-	7buy	Run	input	Done	6 | 32 | 54 - 297	Similar to the previous example, but where the products are protonated.	
-	-	1c0a	Run	input	Done	2 | 1 | 1	https://doi.org/10.1021/acs.jpcb.2c03843	
-	-	3jwe	Run	input	Done	0 | 0 | -		
-	-	3hju	Run	input	Done	3 | 7 | 2		
-	-	5foe	Run	input	Done	3 | 3 | 2		
-	-	1lw4	Run	input	Done	0 | 0 | -		
-	-	5xh3	Run	input	Done	3 | 4 | 2	Substrate trimmed to not include the aromatic rings	
-	-	5xh3	Run	input	Done	0 | 0 | -	Similar to previous one but know including a bigger portion of the substrate.	

+ New From PDB

M-CSA has been funded by EMBL and the Wellcome Trust. It is distributed under a Creative Commons Attribution 4.0 International (CC BY 4.0) License.

2 - Choosing a PDB and Creating a New Search

In order to make a mechanism search, a PDB structure of the enzyme must be chosen containing an active site with the catalytic residues, the substrates, and any co-factors necessary for the reaction. It is not necessary to have these exact molecules in the active site of the selected PDB structure, although a model resembling the native system will yield better results:

- If the catalytic residues are mutated in the PDB structure, the UniProt residue will be considered instead. The 3D structure of the non-mutated residue will be created in the active site and its maximum-common-substructure (MCS) to the mutated 3D residue will be used as guide for its correct positioning.
- Substrates and co-factors not already present in the active site may be added by replacing an existing molecule (including water molecules) in the active site with the desired one. Similarly, the MCS is used to guide the positioning of the native molecule on top of the PDB ligand.

PDB structures of enzymes typically do not contain the wild-type catalytic residues together with the complete set of substrates and co-factors, since that state is labile and hence difficult to capture. We plan to allow the submission of user generated models in the future to overcome this limitation.

At the moment, it is the biological assembly of the chosen PDB structure that is used for the calculation.

After choosing an adequate PDB structure the user can type the PDB code in the input box and click on "New From PDB" to generate a new prediction based on this PDB. After clicking, a new line should appear on the table. The user can then click on "input" to proceed to the next stage.

3 - Choosing the Catalytic Residues

The first step in the input page, after choosing the PDB structure, is to select the catalytic residues involved in the reaction. This can be done by selecting the residues in the drop-down list, and by selecting which part of the residue is involved in the reaction (defaults to side chain) followed by clicking on the "+Residue Button". To speed up the residue selection in the drop-down list, users can also type the name of the residue followed by its residue number.

The drop-down list of residues shows two numbers for each residue. The first is the position of the residue in the PDB chain, while the one after "seq:" is the position of the residue in the protein sequence, as taken from UniProt.

When first selecting a residue, its chain will default to the first one shown in the drop-down list. If it is necessary to change the chain of a particular residue, this can be done by clicking on the appropriate chain in the table of residues. The 3D viewer can be used to make sure all residues are associated with the correct chain (this is particularly important when the active site is situated in the interface of two or more chains). The button "Show Active Centre" will focus on the already selected residues to facilitate this task.

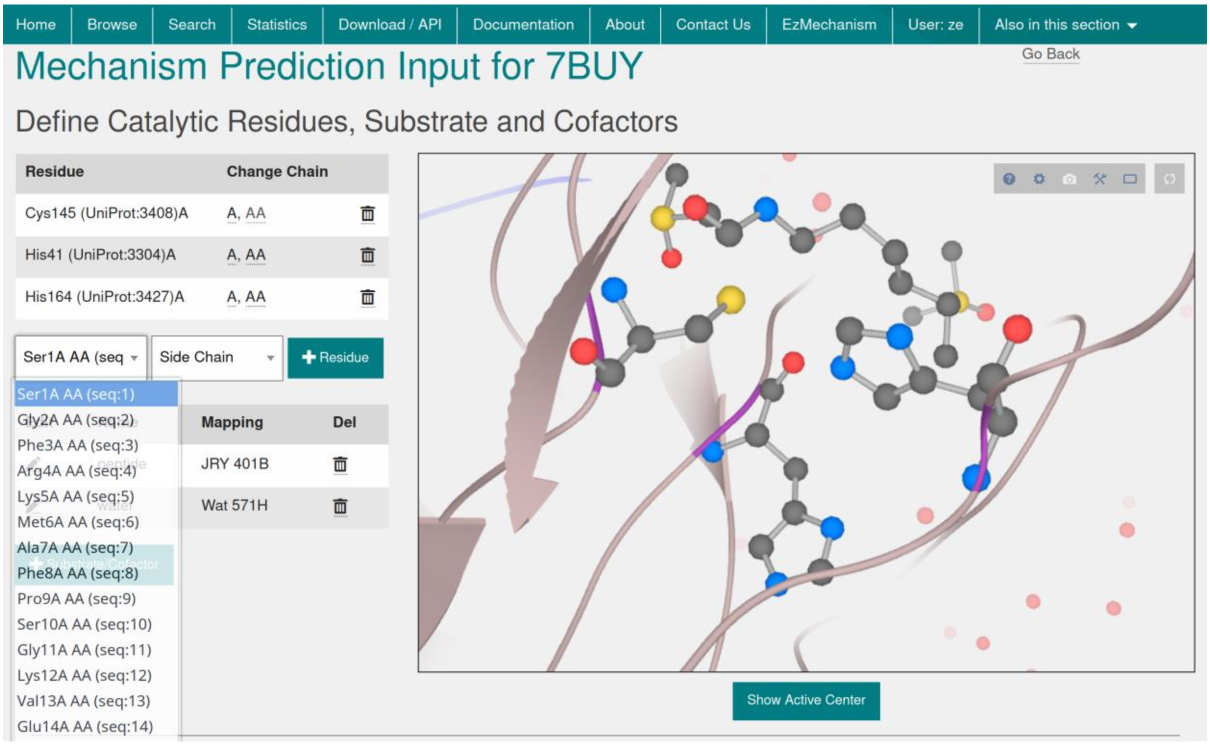

4 - Defining the substrates and co-factors

Substrates and co-factors are treated in the same manner in EzMechanism. To add a new one of these molecules, click on the "+Substrate/Cofactor" button, which will add a new line to the table above. Then click on the edit button (🖉) to access the edit page (figure below).

In the left section of the edit page the molecule can be defined in three ways:

- The user can draw the molecule using the MarvinJS plugin interface.
- If the ChEBI ID of the molecule is known, it can be inserted in the text input box, followed by clicking on the "From ChEBI" button.
- Molecules can be copied and pasted from other MarvinJS instances.

After the molecule is drawn using any of these methods the user needs to click on "Save Mol".

The name of the molecule can be defined in the input text box at the bottom, followed by clicking on "Save Name".

In order to generate appropriate 3D coordinates for the drawn molecule, it needs to be partially mapped to an existing ligand in the PDB structure. On the right side of the page, the user needs to choose to which ligand in the PDB will the drawn molecule be mapped to. The PDB ligands in the drop-down list will be sorted by similarity to the drawn molecule and distance to the active site, so typically the correct ligand should be located towards the beginning of the list.

After selecting a PDB ligand, the maximum-common-substructure between the drawn molecule and the ligand will be shown as green circle highlights. The 3D viewer in the previous page can also be used to confirm the mapping is correct, by comparing the number and name of the residues in the viewer with the selected ones.

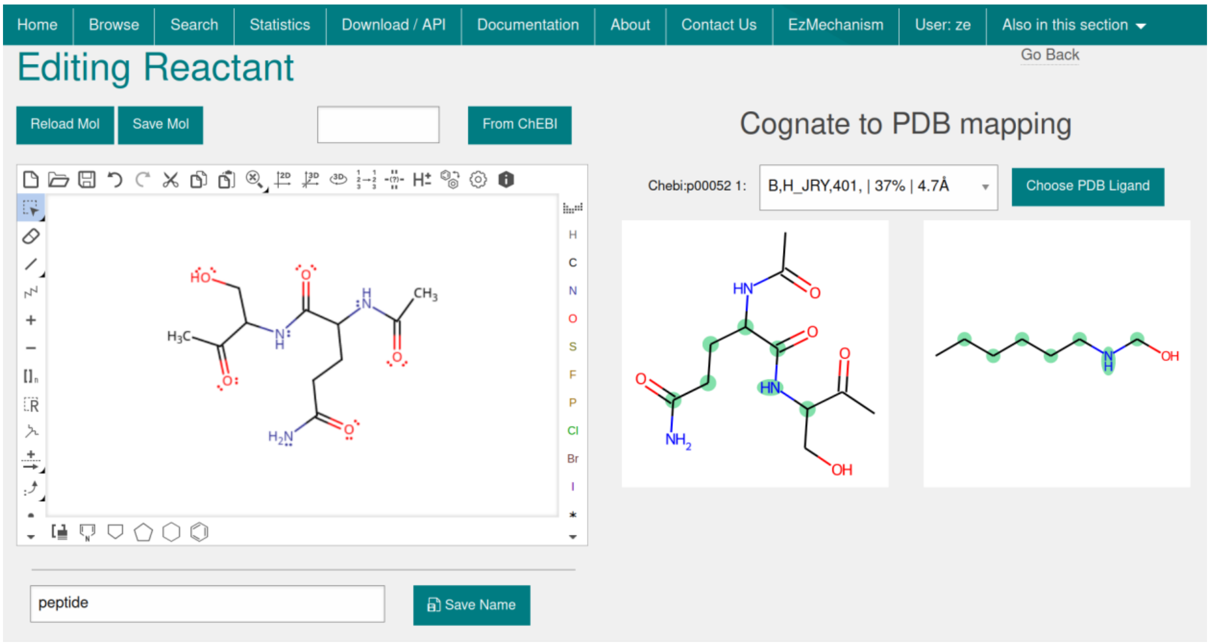

5 - Defining the Overall Chemical Reaction

After defining all the active site molecules, the user needs to specify what is the overall reaction of the enzyme. This is done in the following two MarvinJs boxes (as pictured below), where the one in

the left is used to define the reactants configuration of the active site and the one in the right is used to define the products configuration.

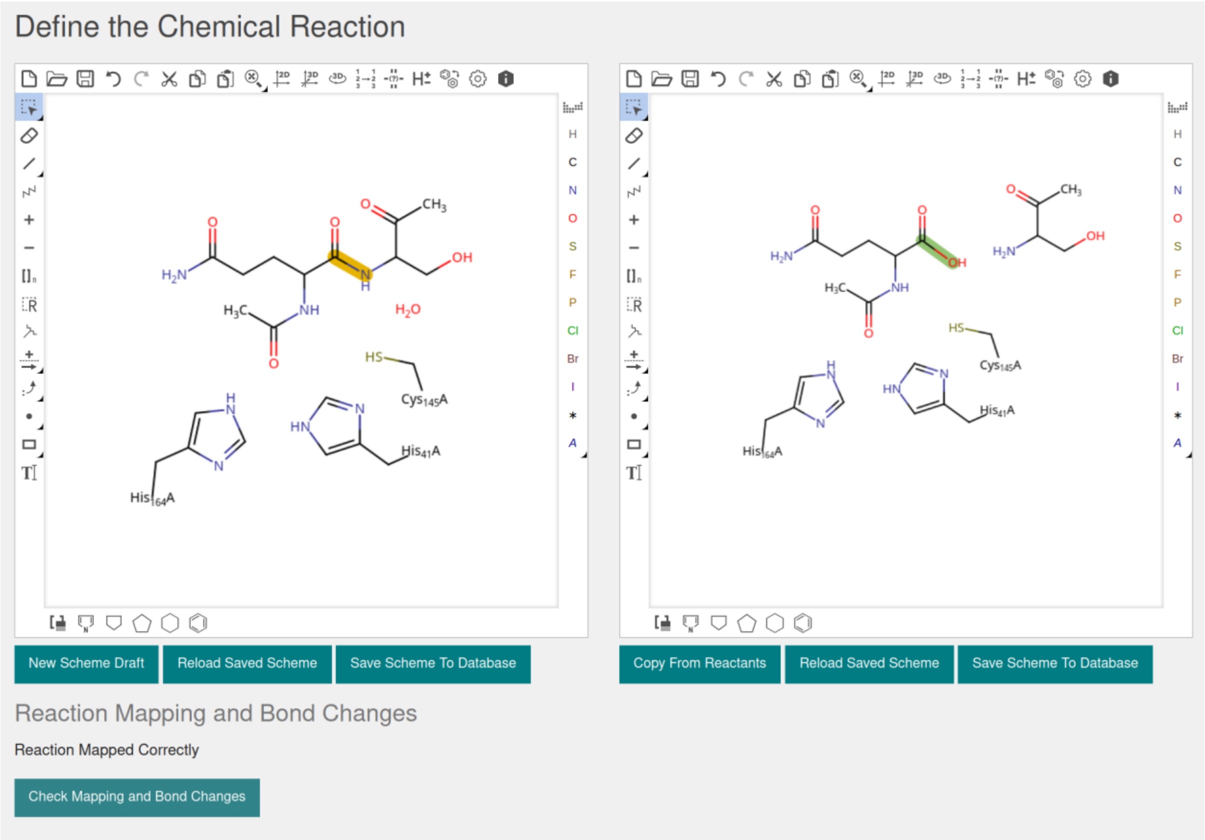

These are the steps necessary to define the overall reaction:

- Click on the "New Scheme Draft" button. This will cause all the molecules involved in the reaction, as defined in the steps above, to appear in the first box. The user can then rearrange the molecules in a disposition that mimics the 3D structure of the active site more closely. The position of the molecules in this 2D scheme does not affect the calculation but can help the analysis of the output.
- The protonation states of the molecules in these diagrams is taken into account during the calculation, so the user must protonate or deprotonate atoms as required. This can be accomplished by using the "+" and "-" buttons to change the charge of the atoms. In order to test alternative protonation states, additional calculations must be performed.
- No new molecules should be added in this viewer since these will not be mapped correctly to the catalytic residues or the defined substrates. However, atoms can be added or deleted to the existing molecules.
- After defining the position of the molecules and their protonation states, click on the "Save Scheme To Database" button.
- To define the products of the reaction, first click on the "Copy From Reactants" button under the box on the right. This will make an exact copy of the reactants configuration as previously defined.
- Using the "Delete" and "Bond" buttons of the MarvinJs box (third and fourth buttons in the left vertical bar, respectively) add and delete bonds as necessary to draw the products configuration. Each atom in the reactants configuration (left) must have a corresponding

atom in the products configuration (product) so if you notice something is missing first draw it in the left box, and then copy the products configuration again. Do not delete or add atoms directly in the right box.

- After defining the products, click on "Save Scheme to Database". To make sure all atoms are mapped correctly between reactants and products, click in "Check Mapping and Bond Changes". This will create an alert for any problems with the mapping and will highlight the cleaved and new bonds in the 2D diagrams.

6 - Defining the Prediction Parameters

Finally, the user defines some calculation parameters. At the moment, the only exposed parameter is the total number of nodes (configurations) the search algorithm checks against the catalytic rules. A bigger number might be required if the mechanism involves a large number of steps or the pose of the 3D structure is not conducive to catalysis. The default number of configurations to explore is 100.

A text box is provided here where the user can write their own comments about the calculation. This field also appears in the overall table that shows all the calculations for the user, so this might be useful to explain what distinguishes this calculation from others.

7 - Submission and Running Status

To submit the search, click on "Run EzMechanism Search" in the input page or on "Run" in the adequate row in the table of the overall EzMechanism page.

The status of the calculation can be tracked in the EzMechanism page. Running calculations will have a red "button" labelled "Cancel" that can be used to interrupt the calculation. The page needs to be refreshed to update the status of the calculation. The figure below shows all the status the calculation will go through before completion.

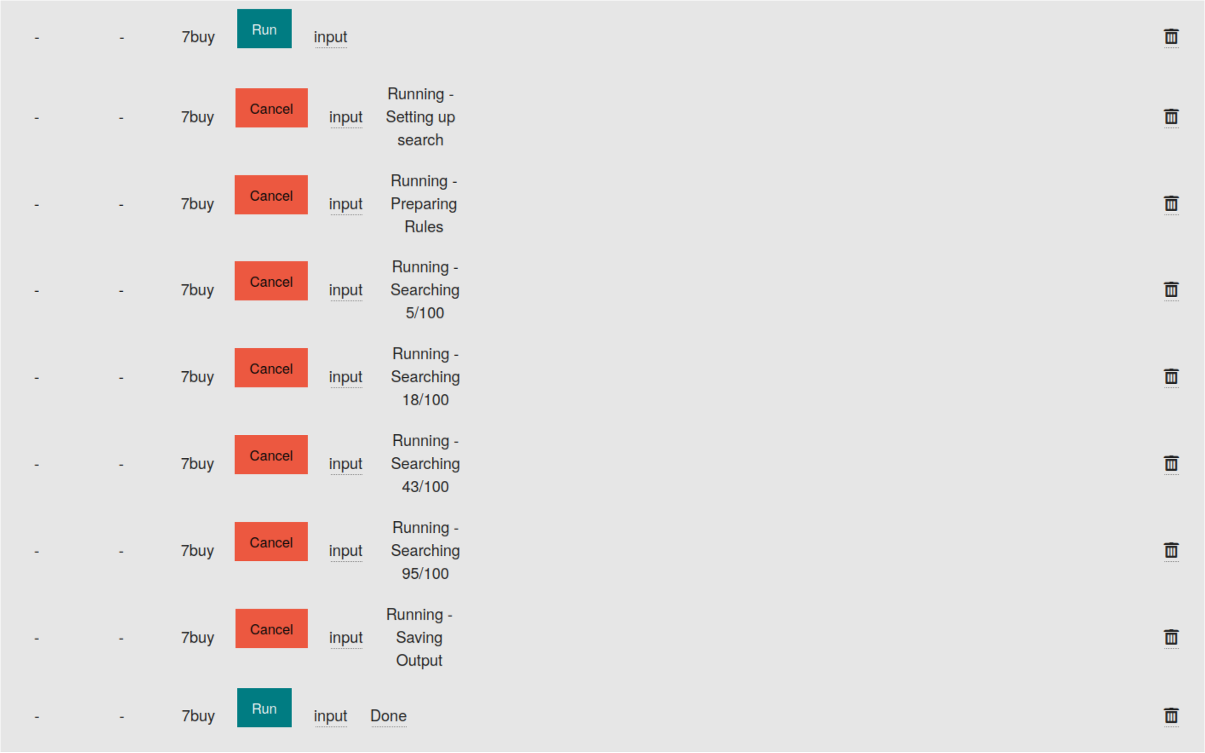

After the calculation is finished, if the user wishes to try a different selection of catalytic residues, protonation states, or parameters, this can be done by opening the input page, change anything as desired, and click on "Run" again. This will delete the results of the previous run. If the user wants to try different parameters while keeping the previous results, a new calculation should be created from scratch.

Description of the Web User Interface – Output Page

The following text and pictures are also available at https://www.ebi.ac.uk/thornton-srv/m-csa/EzMechanism/documentation_output.

The Output Page

The output page of each mechanism search can be accessed by clicking on the "Done" link in the overall EzMechanism table.

The output page contains four (optionally five) main panels. A graph of configurations, steps, and mechanistic paths is shown on the upper left (panel 1). The panel on the upper right (panel 2) contains buttons and sliders used to filter the graph and change its representation. Below the second panel, a third panel shows information about the catalytic rule if any reaction step (edge) is selected in the graph. Panel 4, located below the first three panels shows the 2D diagrams of selected configurations in the graph. Finally, panel 5 is only shown for searches made for enzymes that are already in the database, with the purpose of comparing the annotated mechanism with the results of the search.

In the following sections we explain how to interpret the results of an EzMechanism search and what are the capabilities of the output page.

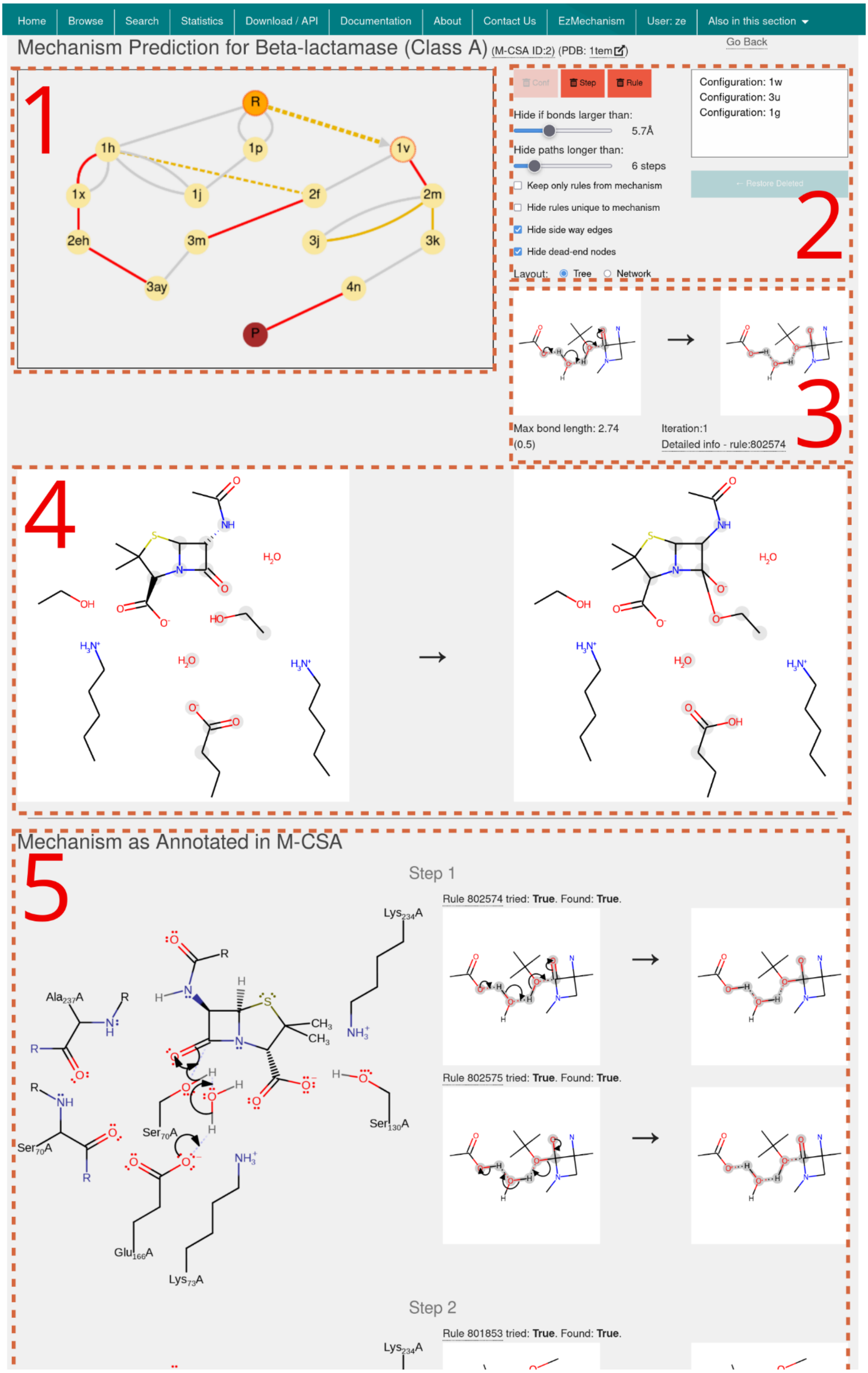

1 - The Results Graph

In the results graph, configurations are represented as circles and reaction steps as the edges that connect two configurations. Each reaction step is associated with a single rule that generated that transformation.

The reactants and products configurations are coloured in orange and red, respectively, and labelled as "R" and "P". Configurations that were matched against the catalytic rules (explored configurations) are shown in yellow, and the remaining configurations, which were generated but not checked against the rules are coloured in grey. The configurations are labelled with a unique identifier that starts with a number representing the distance to the reactants' configuration (in terms of reaction steps), followed by a unique string of letters that hold no particular meaning.

Edges are typically coloured in grey. However, if the prediction is for a mechanism already existing in the database, they are coloured in red if the rule is exclusive to that database mechanism and coloured in orange if the rule is seen in this mechanism but also elsewhere.

2 - Tools to Filter and Manipulate the Results Graph

The purpose of the buttons and slides on the second panel of the output page is to filter and trim the mechanism graph to facilitate comprehension. The three red buttons allow for the removal of any selected configuration, reaction step, or reaction rule (which removes all the reaction steps that follow the selected rule) from the graph. Any elements of the graph deleted in this manner are listed in the neighbouring table and can be restored.

Below the three red buttons there is a slider to filter out any reaction steps that involve the formation of bonds between atoms farther away than the selected cut-off. Note that the distances are based on the position of the atoms in the PDB structure, so the formation of a bond between atoms that are 7 Å away for example, might not be unreasonable, since during the reaction the molecules in the active site might move to bring these atoms closer. A second slider allows the filtering-out of mechanistic paths that are longer that a chosen number of steps. By default, only the shortest paths are shown.

Below the sliders, there are four self-explanatory checkboxes to further limit the number of circles or edges in the graph. The second and third ("keep only rules from mechanism", and "hide rules unique to mechanism") are only relevant when the prediction is for a database entry with a curated mechanism.

Finally, the layout of the graph might be toggled between tree or network-like. The tree layout is particularly useful to see the length of reaction paths, and how far away each configuration is from the reactants. The network-like layout is useful to detect interesting shapes in the topology of the graph, such as bottleneck configurations or reaction steps that are essential to connect the reactants to the products.

3 - Rule Information

When an edge, representing a reaction step, is selected in the results graph, the rule that was used to generate that step is show in the third panel. Also shown, is the largest distance between atoms

that form new bonds in the step, which is used to compute the final prioritization score, given inside parentheses. The iteration number shows how many configurations had to be explored before this reaction step was found. Finally, there is a link to a page that shows detailed information about the rule, including a listing of all the enzymes in the database that follow the rule.

4 - Schemes of the Configurations and Reaction Steps

The two-dimensional scheme of any configuration can be seen in panel 4 if the corresponding circle is selected in the graph. Similarly, if an edge is clicked, two configurations are shown, corresponding to the starting and ending points of that reaction step. In that case, the reaction centres (atoms that are matched to the rules and are involved in bond changes) of the step are highlighted in both schemes. The direction of the reaction step can be reversed for visualisation purposes by clicking a second time on the edge. When an edge is selected, all the edges of reaction steps that follow the same rule have their representation changed to a dashed line.

5 - Comparison with Annotated Mechanism

If these are the results for an EzMechanism search based on an existing entry of the database, the manually curated mechanism is shown in panel 5. The 2D curly arrow diagrams of every step of the mechanism are shown on the left side of the panel, so they can be compared with the output of the prediction. The catalytic rules that were originated from each step are shown on the right, with the indication if those rules were included in the prediction and matched any configuration during the search.

References

1. Ramos-Guzmán, C. A., Ruiz-Pernía, J. J. & Tuñón, I. Unraveling the SARS-CoV-2 Main Protease Mechanism Using Multiscale Methods. *ACS Catal.* **10**, 12544–12554 (2020).
2. Fernandes, H. S., Sousa, S. F. & Cerqueira, N. M. F. S. A. New insights into the catalytic mechanism of the SARS-CoV-2 main protease: an ONIOM QM/MM approach. *Mol Divers* **26**, 1373–1381 (2022).
3. Dutta, S. & Chandra, A. Free Energy Landscape of the Adenylation Reaction of the Aminoacylation Process at the Active Site of Aspartyl tRNA Synthetase. *J. Phys. Chem. B* **126**, 5821–5831 (2022).
4. Galvani, F., Scalvini, L., Rivara, S., Lodola, A. & Mor, M. Mechanistic Modeling of Monoglyceride Lipase Covalent Modification Elucidates the Role of Leaving Group Expulsion and Discriminates Inhibitors with High and Low Potency. *J. Chem. Inf. Model.* **62**, 2771–2787 (2022).

5. Sanz-Martínez, I., García-García, A., Tejero, T., Hurtado-Guerrero, R. & Merino, P. The Essential Role of Water Molecules in the Reaction Mechanism of Protein O-Fucosyltransferase 2. *Angewandte Chemie International Edition* **61**, e202213610 (2022).
6. Rocha, J. F., Sousa, S. F. & Cerqueira, N. M. F. S. A. Computational Studies Devoted to the Catalytic Mechanism of Threonine Aldolase, a Critical Enzyme in the Pharmaceutical Industry to Synthesize β-Hydroxy-α-amino Acids. *ACS Catal.* **12**, 4990–4999 (2022).
7. Zheng, M. *et al.* Computational biotransformation of polyethylene terephthalate by depolymerase: A QM/MM approach. *Journal of Hazardous Materials* **423**, 127017 (2022).
8. Han, X. *et al.* Structural insight into catalytic mechanism of PET hydrolase. *Nat Commun* **8**, 2106 (2017).
